# Estimating the pattern of causes of death in Papua New Guinea

**DOI:** 10.1186/s12889-019-7620-5

**Published:** 2019-10-22

**Authors:** Urarang Kitur, Tim Adair, Ian Riley, Alan D. Lopez

**Affiliations:** 1 Melbourne School of Population and Global Health, University of Melbourne, Melbourne, Carlton, Victoria Australia; 2 National Department of Health, P.O. Box 807, Waigani, National Capital District Papua New Guinea

**Keywords:** Papua New Guinea, Cause of death, Inequalities, Non-communicable disease, Infectious disease, Cause-specific mortality fractions, Emerging diseases

## Abstract

**Background:**

Papua New Guinea (PNG) is a diverse country with high mortality and evidence of increased prevalence of non-communicable diseases (NCDs), but there is no reliable cause of death (COD) data because civil registration is insufficient and routine health data comprise only a small proportion of deaths. This study aims to estimate cause-specific mortality fractions (CSMFs) for five broad groups of causes (endemic infections, emerging infections, endemic NCDs, emerging NCDs and injuries), by sex for each of PNG’s provinces.

**Methods:**

CSMFs are calculated as the average of estimates obtained from: (1) Empirical cause method: Utilising available Verbal Autopsy (VA) data and Discharge Health Information System (DHIS) data, and applying statistical models of community versus facility CODs; and (2) Expected cause patterns method: Utilising existing estimates of mortality levels in each province and statistical models of the relationship between all-cause and cause-specific mortality using Global Burden of Disease (GBD) data.

**Results:**

An estimated 41% of male and 49% of female deaths in PNG are due to infectious, maternal (female only), neonatal and nutritional causes. Furthermore, 45% of male and 42% of female deaths arise from NCDs. Infectious diseases, maternal, neonatal and nutritional conditions account for more than half the deaths in a number of provinces, including lower socioeconomic status provinces of Gulf and Sandaun, while provinces with higher CSMFs from emerging NCDs (e.g. ischemic heart disease, stroke) tend to be those where socioeconomic status is comparatively high (e.g. National Capital District, Western Highlands Province, Manus Province, New Ireland Province and East New Britain Province). Provinces with the highest estimated proportion of deaths from emerging infectious diseases are readily accessible by road and have the highest rates of sexually transmitted infections (STIs), while provinces with the highest CSMFs from endemic infectious, maternal, neonatal and nutritional causes are geographically isolated, have high malaria and high all-cause mortality.

**Conclusions:**

Infectious, maternal, neonatal and nutritional causes continue to be an important COD in PNG, and are likely to be higher than what is estimated by the GBD. Nonetheless, there is evidence of the emergence of NCDs in provinces with higher socioeconomic status. The introduction of routine VA for non-facility deaths should improve COD data quality to support health policy and planning to control both infectious and NCDs.

## Background

Accurate and timely cause of death (COD) data is fundamental for health policy, research and development, but this is lacking in most middle and low-income countries [1–5]. In a low resource, geographically, culturally and socioeconomically diverse country like Papua New Guinea (PNG), understanding COD patterns and differentials in terms of age, sex, ethnicity, socioeconomic status and geography is crucial to identify priority areas for targeted interventions to improve health [6–8]. However, little is known about the distribution of CODs in PNG because the civil registration system does not accurately record death data; reported deaths from health facilities comprise only a small proportion of deaths and there is limited data on the causes of non-hospital deaths [9, 10].

COD data in PNG are available from two sources: (1) publications arising from population-based research and (2) routine health facility-based reporting. Population-based research is limited to small-scale studies in a small number of sites [11–16]. Published data from routine sources are limited to selected hospitals [17, 18] with only two studies reporting on the quality of medical certification of deaths [19, 20]. All of these published studies report infectious diseases as the predominant cause of death in PNG; however recent findings by Gouda et al. [16] and Rarau et al. [21], suggest the emergence of significant levels of non-communicable diseases (NCDs) and NCD risk factors in parts of the country, albeit from sites not representative of the entire PNG population. Estimates from the Global Burden of Disease (GBD) study, suggest NCDs are a major COD in PNG, but these are based on demographic and statistical modelling with very little empirical or local data for PNG, and need to be verified based on empirical evidence [22]. A study by Mola and Kirby showed large discrepancies in maternal mortality ratios (MMR) between modelled estimates and empirical estimates, therefore necessitates the verification of published estimates using local data [23].

The GBD Study classifies CODs under three broad groups: Group I (infectious diseases, maternal, child health and conditions of poverty), Group II (Non-communicable diseases), and Group III (Injuries) [24]. In the PNG context, Gouda et al. [16] further categorised CODs into five groups: (1) endemic infections, (2) emerging infections, (3) endemic NCDs, (4) emerging NCDs, and (5) injuries (see Table 1). This classification divides infections into emerging diseases that are recognised for the first time in the PNG population (e.g., HIV and cervical cancer) or have traditionally affected Papua New Guineans but are re-emerging (such as tuberculosis or TB) and endemic diseases like malaria, pneumonia, diarrhoea, maternal and other infections that have affected PNG’s population for longer [25]. TB is an introduced disease that was thought to be under control in most provinces of PNG in the late 1960s, but the devolution of health services, increased prevalence of diabetes, and drug resistance are factors that are responsible for the current epidemic [26]. HIV was unknown in PNG before 1987 but its emergence thereafter, in combination with TB, has resulted in a substantial number of hospital deaths among young adults [27]. Malaria and pneumonia have likely been leading CODs in PNG in the last 50 years; however, deaths from both diseases appear to have declined [28, 13].

**Table 1:** Composition of five broad COD groups [16]

Group	Cause (ICD-10 code)	ICD-10 code
1. Endemic Infections	Malaria	[B50-B54]
	Diarrhoea/Dysentery	[A00-A09]
	Pneumonia	[J10-J22, J85]
	Maternal	[O00-O99]
	Other infectious diseases^a^	
2. Emerging Infections	TB	[A15–19]
	HIV	[B20-B24]
	Cervical cancer	[C53]
3. Endemic NCDs	Chronic respiratory diseases	[J40-J46]
	Leukaemia/Lymphoma	[C81-C85; C91-C96]
	Cirrhosis	[K70–76]
	Renal failure	[N17-N19]
	Breast cancer	[C50]
	Stomach cancer	[C16]
	Oesophageal cancer	[C15]
	Colorectal cancer	[C18–21]
	Prostate cancer	[C61]
	Other NCDs^a^	
	Other cancers^a^	
	Other cardiovascular diseases^a^	
4. Emerging NCDs	Diabetes	[E10-E14]
	Stroke	[I60-I69]
	Ischemic heart diseases	[I20-I25]
	Lung cancer	[C34]
5. Injuries	Homicide	[X85-Y09]
	Falls	[W00-W19]
	Road traffic	[V01-V89]
	Drowning	[W65-W74]
	Fires	[X00-X19]
	Bite of venomous animal	[X20–29]
	Poisonings	[X40–49]
	Suicide	[X60-X84]
	Other injuries^a^	

^a^ Calculated as the difference between total deaths within that sub-category and the total of specific causes within that sub-category

**Table 2:** Estimated CSMFs (%) by age and sex, empirical cause method, expected cause pattern method and final estimates, PNG, 2011

Method	Disease Classification	Male (%)					Female (%)				
		< 5	5–44	45–64	65+	All	< 5	5–44	45–64	65+	All
Empirical cause method	Endemic Infections	84	27	25	24	36	85	36	22	24	39
	Emerging Infections	2	20	19	5	12	3	22	19	5	13
	Endemic NCDs	10	18	32	43	28	10	21	34	42	28
	Emerging NCDs	0	12	13	16	11	0	8	14	16	10
	Injuries	4	22	11	12	13	3	12	11	14	10
	*Total*	*100*	*100*	*100*	*100*	*100*	*100*	*100*	*100*	*100*	*100*
Expected cause patterns method	Endemic Infections	81	20	12	12	27	80	33	14	12	32
	Emerging Infections	2	13	11	5	8	3	27	17	5	15
	Endemic NCDs	12	24	36	39	30	13	22	35	36	27
	Emerging NCDs	0	8	30	39	22	0	6	30	44	20
	Injuries	5	35	11	5	14	4	13	5	3	7
	*Total*	*100*	*100*	*100*	*100*	*100*	*100*	*100*	*100*	*100*	*100*
Final estimates	Endemic Infections	82	24	19	18	31	82	35	18	18	35
	Emerging Infections	2	17	15	5	10	3	25	18	5	14
	Endemic NCDs	11	21	34	41	29	12	22	35	39	27
	Emerging NCDs	0	10	21	27	16	0	7	22	30	15
	Injuries	4	28	11	8	13	3	12	8	8	8
	*Total*	*100*	*100*	*100*	*100*	*100*	*100*	*100*	*100*	*100*	*100*

Emerging NCDs include diabetes mellitus, stroke, ischaemic heart disease (IHD) and lung cancer, diseases that are largely attributable to individual lifestyle choices, while endemic NCDs comprise chronic respiratory diseases, other cancers, other cardiovascular diseases and all other NCDS. Chronic respiratory diseases among adults are thought to be common in PNG due more to domestic pollution from burning wood in most traditional societies of PNG than from smoking [29, 30]. Diabetes and stroke appear to be increasing in most urban and peri-urban societies of PNG due to life style changes from traditional diets to processed foods, physical inactivity and high rates of cigarette smoking [21]. In this study, we have used Gouda et al’s classification to further categorise diseases into these broad categories within GBD Groups I and II since doing so is potentially more informative for public health policy [16].

Given the limited availability of data in PNG, there is enormous uncertainty in COD estimates. This study aims to provide more reliable estimates by generating cause-specific mortality fractions (CSMFs) for five broad groups of causes, by sex, and for each of PNG’s provinces utilising a statistical modelling framework that encompasses available verbal autopsy (VA) derived COD data for community (non-facility) deaths from the Gouda et al. study [16] and National Department of Health (NDoH) Discharge Health Information System (DHIS) routine facility-based data, the GBD database of COD and all-cause mortality estimates, and existing estimates of all-cause mortality by age, sex and province in PNG [10, 31]. This approach thus consolidated empirical evidence on COD and all-cause mortality with expected cause patterns, given the level of all-cause mortality and sociodemographic development in the country to yield COD estimates at national and provincial levels.

## Methods

### Data sources

#### Facility deaths

The most comprehensive source of facility deaths in PNG is the NDOH Discharge Health Information System (DHIS). Set up in 1968, the DHIS reports deaths from 20 provincial hospitals, 635 health centres and clinics out of 755 registered health facilities (86% reporting rate) in the country [32]. DHIS deaths are those who died in the facilities, except for those who die on arrival, which are regarded as coroner’s cases. Only a very small proportion of deaths are of people residing out of the province, except for the four regional hospitals of Port Moresby in the National Capital District, Mt Hagen in Western Highlands Province (WHP/Jiwaka), Angau in Morobe Province and Nonga in East New Britain Province (ENBP). Deaths in hospitals and health centres are recorded on the standard international medical certificate, with information on age, sex, facility/district/province and COD transferred into the DHIS. Data on deaths in the DHIS are reported using the PNG 3-digit shortlist version of the International Classification of Diseases – Tenth Revision (ICD-10), for over 300 causes, and with age recorded for all deaths. Limitations of the DHIS, detailed elsewhere, are that it is unsuitable for population level mortality because of its exclusion of deaths outside facilities, and that reporting of deaths in some facilities is incomplete in some years. This study uses DHIS data from 2007 to 2013 [32]. The other NDOH data source, the National Health Information System (NHIS), records more deaths than DHIS but only reports deaths based on 26 syndromes. Moreover, 72% of deaths do not have an age recorded, greatly limiting their analytical and policy utility. A new data source, the eNHIS, developed in 2014 records facility deaths using detailed ICD-10 coding but currently operates only in eight provinces, and is still in the early stages of development, with limited numbers of death. Neither NHIS nor eNHIS data were used for analysis in this study given these limitations.

#### Community deaths

The only source of data on community CODs in PNG are from four sites in the Gouda et al. study [16]. From 2009 to 2014, 1408 community and hospital deaths were recorded from the sites and diagnosed using Smart Verbal Autopsy (the Population Health Metrics Research Consortium (PHMRC) Tariff v.2.0 method). Verbal autopsy is a means of obtaining the probable COD based on signs and symptoms reported in a standardised interview with a family member of the deceased [33]. The Tariff algorithm estimates the most probable COD from a list of 32 specific causes for adults. Three of the study sites (West Hiri in Central Province, Asaro in Eastern Highlands Province and Karkar in Madang Province) are in the top 20 districts in terms of socioeconomic development and access to health care as measured by a composite index (described below), while the other site, Hides (Southern Highlands/Hela), is towards the bottom [ 10, 31, 34].

#### Global burden of disease study

The GBD Study provides estimates of all-cause mortality and CSMFs by detailed age group and sex for 195 countries and territories for each year 1990–2017 [35]. The GBD uses the statistical modelling framework Cause of Death Ensemble model (CODEm), which combines results from global statistical models of 192 causes of death. For data-scarce countries like PNG, these models are based primarily on covariates. Covariates include the socio-demographic index (SDI), which is the geometric average of education, economic and fertility indicators and is measured for every country-year, and available risk factor data (e.g. cigarette consumption for lung cancer) [36].

### Analytical methods

Given the potential biases and measurement uncertainty associated with extrapolating from the VA samples and also the local imprecision arising from using global models of mortality developed by the GBD to estimate COD patterns in PNG, we had no a priori basis to favour one method over the other and hence the final estimates of COD suggested by this study were calculated as the simple arithmetic average of estimates obtained from two methods:

1. Empirical method: Utilising available data (VA data, DHIS) with statistical models of community compared with facility CODs.
2. Expected cause patterns method: Utilising existing estimates of mortality levels in each province with statistical models of the relationship between all-cause and cause-specific mortality generated from GBD data.

This approach makes use of the available data, with all their limitations, but also draws on what might be the expected cause patterns given the level of all-cause mortality in each province. CSMFs were estimated for the year 2011, which is close to the mid-point year of the DHIS and VA data and was used for the all-cause mortality estimates applied in this analysis [10].

The basic cause of death measure estimated was the CSMF, defined as the fraction of deaths in the population that is due to each cause. CSMFs were calculated for four broad age groups across which the composition of the leading causes of death was likely to change (0–4, 5–44, 45–64, 65+ years), and for each sex and each province. The four age groups were chosen because leading causes of death commonly vary between each age group; more detailed age groups could not be used because of the limited data available from VA.

Causes of death were first estimated for the five cause categories as defined by Gouda et al.: namely emerging infections, endemic infections, emerging NCDs, endemic NCDs, and injuries [16]. Table 1 lists the main specific causes included under each category. A more precise cause listing was not possible again because of the limited data available from VA.

#### Empirical cause method

In the empirical cause approach, we estimated CODs separately for facility and community deaths. Facility deaths are defined as those reported by the DHIS and community deaths are defined as all other deaths. Thirty-eight thousand three hundred three DHIS deaths recorded from 2007 to 13 were used to calculate facility-based CSMFs. Deaths recorded in all these years were used due to the low numbers of deaths in some provinces, especially at older ages, in order to reduce random error. The most recent year of data available is 2013.

Community CSMFs were estimated for each province as follows. For each of the four sites with VA data, the ratio of community CSMFs (from VA) to DHIS-derived CSMFs was calculated, in log space, for each age group and sex. These ratios were then applied to the DHIS CSMFs in each of the provinces to estimate the community CSMFs by age and sex. This method assumes that the ratio of community CSMFs to DHIS CSMFs (within each age and sex group) is constant across provinces. Further detail about the method employed is presented in the Additional file 1.

Once community and DHIS CSMFs were calculated, CSMFs for all deaths by age, sex and province were calculated by weighting them by the proportion of all deaths that occurred within and outside facilities. The proportion of all deaths that occurred within and outside facilities was calculated, for each age, sex and province, as DHIS deaths divided by total deaths, based on the province-specific life tables calculated by Kitur et al. and provincial population data [10]. This method is described in detail in the Additional file 1.

#### Expected cause patterns method

The expected cause patterns approach uses data from the GBD 2017 study. The GBD data were used to develop linear regressions, for each of the four age groups, with outcome variable of the natural log of the ratio of each specific cause to a base cause (endemic NCDs) and covariates of the natural log of the probability of dying in that age group (using the all-cause mortality estimates of Kitur et al), calendar year and SDI [10]. This regression was developed for each sex and cause and these were used to predict CSMFs at the national level for PNG. Provincial-specific estimates were made based on regressions as above but excluding the SDI measure because there is no equivalent available for PNG’s provinces. These CSMFs were then predicted for each province, age, sex and then scaled to the national level CSMFs in PNG. More details about this method can be found in the Additional file 1.

#### Ethics and data permission

We used aggregated cause of death (COD) data from a published PNGIMR study [16], publicly available Global Burden of Disease data and from the National Department of Health where the lead author works. Since these data sources contained aggregated data with no personal identifiers and no risk to any individual, this was considered a low risk research that required no ethical approval. Verbal approval was granted from NDoH in 2016 to use the DHIS data.

#### Presentation of findings

The findings are presented in the main body of the text and the Additional file 1 for each broad age group, sex and province. The plausibility of CSMF estimates and patterns of geographical distribution were assessed by comparing provincial CSMFs to a composite index developed by Kitur et al. [10, 31] which measures provincial differences in socioeconomic development and health access. The composite index is derived from the arithmetic mean of education, economic, and health access indicators, with each indicator adjusted to be a normally distributed percentage with a mean of 50%. The education indicator measures net admission rate (percentage of children aged 6 years who are admitted into elementary prep school) and female literacy rate while the economic indicator is an average of poverty levels as assessed by the World Bank based on food and non-food expenditure and the proportion of people engaged in paid work activities from the 2011 census. The health access index was computed based on information about number of health workers per population and immunization rates from the 2011 Health Sector Performance Annual Review [34, 37, 38].

## Results

### National level cause specific mortality fractions (CSMFs)

In Table 2, the empirical cause method suggests a higher proportion of deaths from infectious diseases, maternal (females only), child health and conditions of poverty and a lower proportion from NCDs, compared with the expected cause patterns method, at all ages and in both males and females. Injury cause-fractions are higher in the empirical cause method at all ages except 5–44 years. More detailed information is provided in the Additional file 1: Table S1. These data show that there are more facility deaths from infections in children under 5 years than in the community and consistently higher injury deaths in the community.

Nationally, at all ages, more females (49%) die from infectious, maternal, neonatal and nutritional conditions (Groups 1 & 2) than males (41%). Conversely, slightly more males (45%) than females (42%) die from NCDs (Groups 3 & 4). Groups 1 & 2 CSMFs decline with age, and at ages 5–44 are 60% of female deaths, with emerging infections (Group 2) comprising 25%. NCDs, particularly emerging NCDs (Group 4), increase with age and account for 27% of male deaths and 30% of female deaths at ages 65+ years. Overall, endemic diseases (Groups 1 & 3) comprise over half of all deaths and emerging diseases (Groups 2 & 4) just over one-quarter. At ages 45–64 years, emerging diseases comprise 36% of deaths in males (15% emerging infections, 21% emerging NCDs) and 40% of deaths in females (18% emerging infections, 22% emerging NCDs). Injury CSMFs at ages 5–44 years in males (28%) are more than double than in females (12%).

### Provincial estimated cause fractions by sex

CSMF estimates for the different disease groups vary considerably by province (Table 3). We present results primarily for both sexes, but identify sex-specific differences of note. Additional file 1: Table S2 presents detail information on sex-specific mortality differences.

1. *Endemic infections (malaria, pneumonia, diarrhoea), neonatal, nutritional and maternal*

**Table 3:** Estimated CSMFs for both sexes (%), under-five mortality (per 1000 live births) by sex, life expectancy (years) by sex and composite index (%) by province and sex, PNG, 2011^a^

Province	Endemic Infections	Emerging Infections	Endemic NCDs	Emerging NCDs	Injuries	Under 5 mortality per 1000		Life expectancy in years		Composite index (%)
	Both sexes (%)					Male	Female	Male	Female	
*PNG*	*32.7*	*11.7*	*28.1*	*15.6*	*10.8*	*68*	*58*	*62.0*	*64.3*	*50*
National Capital District	25.2	12.8	30.0	23.0	9.4	34	23	67.0	70.3	95
East New Britain	34.9	10.4	27.6	16.9	9.6	65	56	62.2	63.9	75
New Ireland	33.9	8.7	31.1	16.0	9.3	62	48	63.8	65.9	60
Bougainville	35.8	9.8	27.4	14.4	11.5	54	43	62.4	67.2	60
Eastern Highlands	28.4	11.9	30.6	15.6	14.1	53	45	64.9	64.4	59
Morobe	36.9	13.9	26.6	13.0	10.1	84	71	59.7	61.0	59
Western Highlands/Jiwaka^b^	23.6	10.6	35.2	18.6	12.4	40	33	65.9	68.7	58
Simbu	25.0	15.4	31.0	17.0	11.0	40	35	67.2	64.2	57
West New Britain	37.3	12.3	22.6	14.0	13.3	54	45	63.4	65.2	56
Milne Bay	40.4	8.8	28.0	14.0	8.7	78	64	61.5	65.4	51
Manus	35.5	6.8	31.5	17.5	8.1	83	75	62.6	64.4	50
East Sepik	38.3	9.8	25.4	13.0	13.9	100	79	58.8	62.6	49
Madang	37.2	13.3	25.6	12.7	10.8	78	69	60.2	62.4	48
Central	28.8	8.3	29.0	17.1	16.8	55	44	62.4	63.8	45
Oro	35.7	11.9	29.7	14.6	8.7	69	64	62.0	64.6	43
Western	36.2	9.4	29.4	13.6	11.3	64	57	61.8	67.2	41
Sandaun^c^	43.4	12.4	23.5	10.1	10.7	131	119	54.4	56.8	29
Southern Highlands /Hela^b^	34.8	12.4	28.6	13.0	10.9	70	59	59.6	65.4	28
Gulf	41.4	13.5	22.5	10.0	12.1	109	97	56.2	57.4	27
Enga	25.0	13.6	29.1	14.8	17.4	69	66	60.7	62.0	25

^a^This table has been sorted by the composite index ranking (%), from the highest rank in level of socioeconomic development and health access (National Capital District) to the lowest rank (Enga Province). ^b^ These provinces were still united at the time of data collection. ^c^ Sandaun Province is also known as West Sepik Province

The proportion of deaths from endemic infectious, neonatal, nutritional and maternal causes (females only) are highest in Sandaun Province (43%), Gulf Province (41%), Milne Bay Province (MBP) (40%), East Sepik Province (ESP) (38%) and Morobe Province (37%). All these provinces have high levels of all-cause mortality as shown Additional file 1: Figure S1-S3.

2. *Emerging infections (TB, HIV, cervical cancer)*

CSMFs from emerging infections are highest in the highlands of Simbu Province (15%), Enga Province (14%), Southern Highlands Province (SHP/Hela 12%) and Eastern Highlands Province (EHP) (12%), in Morobe Province (14%) and Madang Province (13%) and the National Capital District (NCD). Apart from the National Capital District, all these provinces are linked by the Highlands highway and report the highest rates of sexually transmitted infections (STIs) in the country [39].

3. *Endemic NCDs (chronic respiratory diseases, cancers, renal failure, liver cirrhosis)*

A large proportion of deaths from endemic NCDs are from the central highlands provinces of WHP/Jiwaka (35%), Simbu Province (31%), and EHP (31%); Manus Province (32%) and New Ireland Province (NIP) (31%) and National Capital District (30%). The lowest proportions of deaths from endemic NCDs are found in Gulf Province and Sandaun Province, although there is relatively little variation amongst provinces in this cause.

4. *Emerging NCDs (diabetes, stroke, ischaemic heart diseases, lung cancer)*

Provinces with the highest cause fractions for emerging NCDs include National Capital District (23%) and Central Province (17%), WHP)/Jiwaka (19%), Simbu Province (17%), and EHP (16%) and Manus Province (18%), ENBP (17%) and NIP (16%). The lowest CSMFs are found in Gulf Province and Sandaun Province, at approximately 10%. The proportion of deaths from emerging NCDs is positively related with a province’s level of life expectancy. WHP/Jiwaka, Simbu Province, National Capital District, Manus Province and NIP report the highest rates of deaths from NCDs among males and females, at about 50% of deaths for males and slightly lower for females.

5. *Injuries (homicide, falls, road traffic accidents, bites of venomous animals, fires, poisoning, suicide)*

CSMFs for injury deaths are highest in Enga Province (17%), Central Province (17%), EHP (14%), ESP (14%) and West New Britain Province (WNBP) (13%). These provinces have also been found to have the highest rates of violence, road traffic accidents and falls [16, 43, 44].

### Relationship of cause fractions with under five mortality and life expectancy

NCDs, especially emerging are higher in low mortality provinces and infections are higher in high mortality provinces. Table 3 shows great variation in mortality between provinces with the lowest (National Capital District, male 34/1000, female 23/1000) and highest (Sandaun Province male 131/1000, female 119/1000) level of child mortality estimates. In both sexes, there is a life expectancy gap of about 13 years between the province with the lowest levels (Sandaun Province; male 54.4 years, female 56.8 years) and the National Capital District (male 67.0 years, female 70.3 years), the province with the highest life expectancy in PNG.

### Composite index

**Fig. 1:**
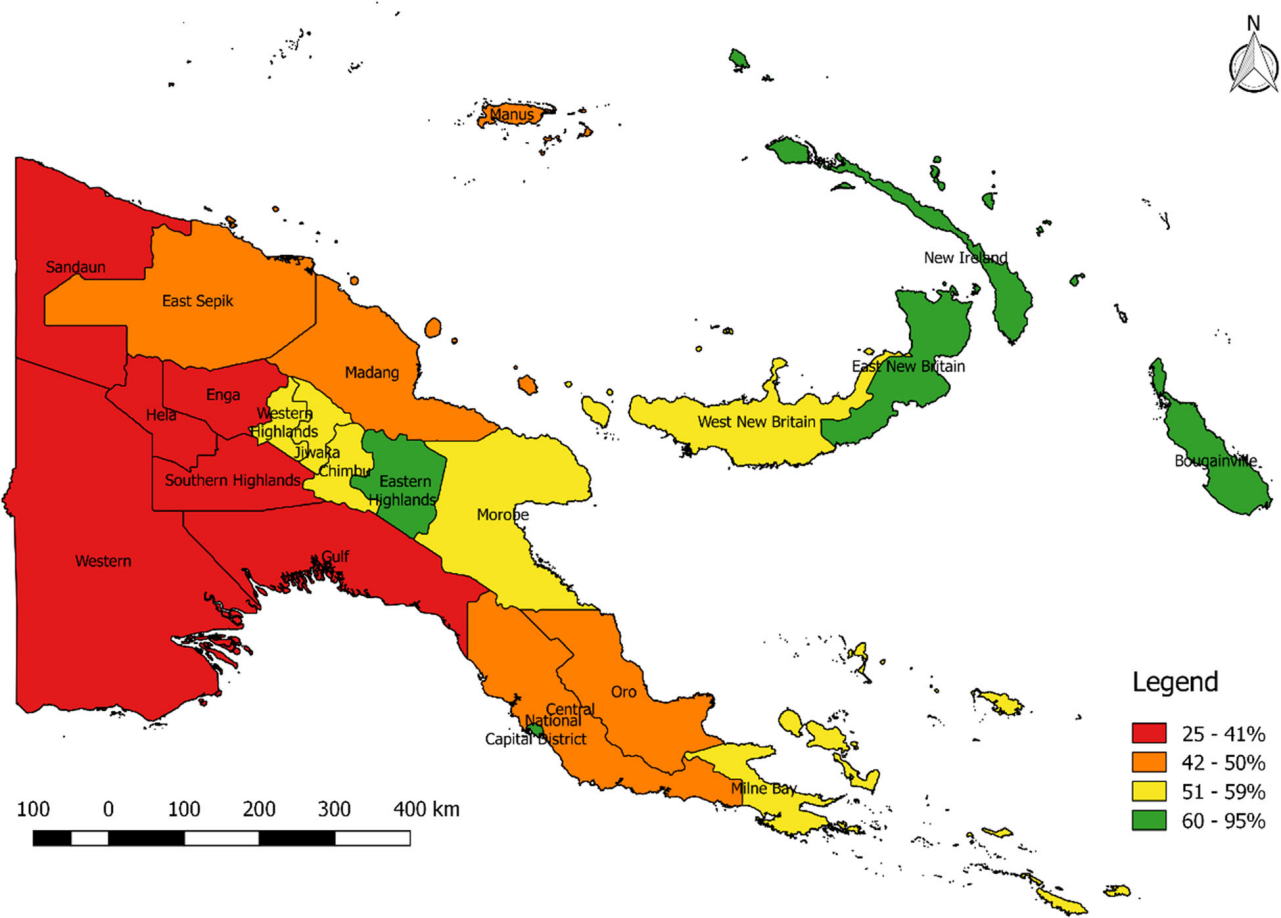
Composite index [10] by province, PNG. The legend depicts the ranking of socioeconomic development (%) by province, with the more developed provinces ranking higher than less developed provinces. The legend displays red, orange, yellow and green colours by provinces according to the level of development. Red colour displays provinces with low levels of development, orange shows low to moderate, yellow shows moderate and green shows high level of development as ranked by the composite index

The level of socioeconomic development and health access varies significantly across PNG as shown in Table 3 and Fig. 1. Development is low in the western (Sandaun Province, Enga Province and Western Province) and southern (SHP/Hela, Gulf Province, Central Province and Oro Province) and comparatively high in the National Capital District and in East and West New Britain, NIP and Bougainville. It is moderate to high in the central highlands provinces of WHP/Jiwaka, Simbu Province and EHP, while in the north (Morobe Province, Madang Province and ESP the composite index is low to moderate. More information is available in the Additional file 1: Table S3.

**Fig. 2:**
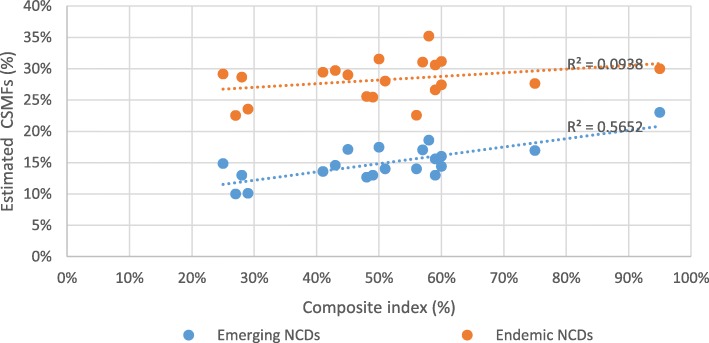
displays the relationship between provincial Composite index [10] and provincial CSMFs for endemic and emerging NCDs of both sexes in PNG, 2011

**Fig. 3:**
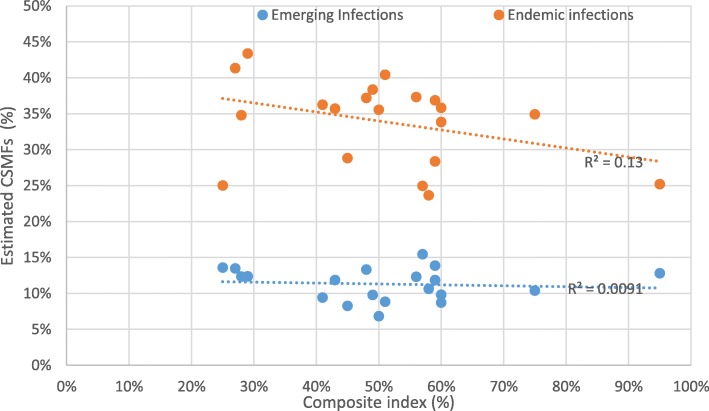
shows the relationship between provincial Composite index [10] and provincial CSMFs for endemic and emerging infections in both sexes, in PNG 2011

The provincial composite index correlates strongly with CSMFs for both sexes from emerging NCDs (Fig. 2), but less so with other causes (Figs. 2 and 3). There are no clear outliers among any provinces in the relationship between the composite index and emerging NCDs, with a reasonably strong r-squared of 0.57. The weakness of the relationship between the composite index and endemic NCDs (r^2^ = 0.09), emerging infectious diseases (r^2^ = 0.01), and endemic infectious diseases (r^2^ = 0.13) is confirmed by their low r-squared.

## Discussion

Our findings demonstrate that infectious, maternal (females only), neonatal and nutritional causes (Groups 1 & 2) are still an important cause of death in PNG, accounting for more than 50% of all deaths in some provinces. However, NCDs (Groups 3 & 4), especially emerging NCDs (Group 4), are particularly high in provinces with higher levels of socioeconomic development. These results demonstrate the challenge for public health policymakers in PNG in addressing the dual burden of infectious diseases and NCDs, particularly with significant mortality occurring due to emerging forms of each.

Our findings from this study show Groups 1 & 2, both in children and also adults and are much more important as a cause of death accounting for 41% of deaths in males and 49% of deaths in females. This is almost double what is reported by the GBD [35] (23% for males and 29% for females) and for other Melanesian countries [40, 41]. The GBD Study estimates lower infectious disease mortality for PNG due to the greater reliance on data from neighbouring countries such as Fiji, Tonga and Samoa, all Pacific countries with higher NCD mortality [35]. Our findings also show that more boys than girls under the age of five years die at health facilities which is not uncommon. This is well documented in the literature and is due both to boys’ biological frailty and their higher accident mortality. For example, the UN (UNIGME, 2013) have estimated that for every 1000 live births in 2012, there were 50 male deaths compared to 46 female deaths in children under 5 years globally [42]. According to the same report, there were 68 male deaths compared to only 58 female deaths per 1000 live births under the age of five in PNG, and is consistent with estimates published in an earlier article [10].

### Endemic and emerging infectious diseases, maternal, neonatal & nutritional causes (groups 1 & 2)

Groups 1 & 2 still account for more deaths than deaths from NCDs in most provinces of PNG. Mortality from this broad group of causes are affected by geography and interprovincial movement and does not depend so much on socioeconomic development, as our findings show. The largest proportion of deaths from endemic infectious diseases and maternal causes (in females) occur in Sandaun Province, Gulf Province, ESP and Milne Bay Province; provinces that are relatively remote, report high malaria cases and have the highest all-cause mortality in PNG [10, 28]. Deaths from emerging infectious diseases are highest in Simbu Province, Enga Province, EHP, Morobe Province and Madang Province; provinces that are accessible by the Highlands highway and National Capital District. These provinces report the highest rates of sexually transmitted infections, including HIV and human papilloma virus (HPV) which are responsible for deaths from AIDS and cervical cancer in PNG [39]. High mortality from emerging infections can be directly linked to the epidemic of HIV, to synergism between TB and HIV, and cervical cancer to Human Papilloma Virus.

### Endemic and emerging NCDs (groups 3 & 4)

Non-communicable diseases are becoming more prominent than infectious diseases, maternal and conditions of poverty globally [35], regionally [40, 41], and in some sections of the PNG population [16, 21]. Our findings demonstrate that evidence of more advanced epidemiological transition in PNG, measured both as the % of all deaths from but also in comparison to the level of infectious disease mortality, is related to higher socioeconomic status [23]. National Capital District, WHP/Jiwaka, Simbu Province, EHP, Manus Province and NIP have relatively higher life expectancy. Emerging NCDs, such as IHD, stroke and diabetes, are becoming particularly prominent in these provinces with higher socio-economic development, demonstrating progress through the epidemiological transition and further supporting the claims by Gouda et al. [16] that the transition is not uniformly distributed across PNG. The three central Highlands provinces of WHP/Jiwaka, Simbu and EHP have experienced modernisation through small-scale commercial enterprise, fresh food and cash cropping, and through access to goods and services in Mt Hagen, Kundiawa and Goroka [43]. Port Moresby is a relatively developed city that is accessible by the majority of the population of Central Province (which also has high emerging NCD) who live in peri urban areas of the capital city. Furthermore, changes in lifestyle in Manus Province, NIP, ENBP, National Capital District and Central Province (particularly western parts) including high rates of cigarette smoking, inactivity and unhealthy diets are responsible for high rates of diabetes and coronary heart diseases in these provinces [21].

Males account for twice the burden of injuries as females, similar to what has been reported in Vanuatu [40]. Provinces with the highest burden of injuries are believed to have the highest rates of violence and road traffic accidents. Enga province, ESP and WNBP have the highest rates of violence-related deaths in PNG [44], while EHP and Central Province record more deaths resulting from road traffic accidents [45]. Gouda et al. reported homicide as the major cause of injuries in the highlands (Asaro and Hides), while falls from buildings was largely responsible for the majority of deaths from injuries in the coastal areas (Hiri and Karkar) [16].

### Limitations

There are a number of limitations in our study. Firstly, the empirical cause model was developed using VA and DHIS data from only four sites and applied to DHIS data throughout the country; the use of community cause data from only four sites was necessary due to the unavailability of other data. Hence, we also used the expected cause pattern model, to offset any implausible provincial level results. Secondly, the diagnostic accuracy of the DHIS data has yet to be established, however the use of five broad cause groups reduces any potential error from this issue. Thirdly, Port Moresby General Hospital could have included deaths from people travelling in from other provinces including Central and Gulf. Hence, deaths recorded under Port Moresby General Hospital could not exclude those residing outside of Port Moresby. The same could be said of other regional hospitals including Mt Hagen in WHP/Jiwaka and Angau in Morobe Province. Fourthly, our estimated cause fractions are not as specific as those produced by GBD and hence potentially less useful for policy. However, we were unable to reliably conclude what might have been the cause of death distribution at a more granular level from the available data. Nonetheless, understanding where various provinces might be on the path of epidemiological transition, and particularly the likely extent of emerging diseases as causes of death has, we believe, intrinsic policy value. Finally, we were also unable to estimate the level of uncertainty around our CSMF estimates in this study due to the unavailability of data. Based on a comparison with the GBD modelled estimates for PNG, our estimated CSMFs generally fell within 20% of these predicted estimates, which are themselves subject to significant uncertainty because of a lack of data in the country.

## Conclusion

Our estimated cause-specific mortality fractions are the first such estimates to be based on empirical data for PNG and its provinces. While infectious diseases, maternal, neonatal and nutritional causes remain an important cause of death in PNG; there is evidence of the emergence of NCDs as leading cause of death in the highlands provinces of WHP/Jiwaka and Simbu, islands provinces of NIP, ENBP and Manus and in National Capital District, consistent with their higher level of socioeconomic status. CSMFs from emerging infections are influenced by high rates of STIs in provinces that are linked by road while CSMFs from endemic infections are related to malaria endemicity in geographically isolated provinces. While socioeconomic development has been shown to explain epidemiological transition (emerging NCDs), geographical and cultural factors are also responsible contributors to CSMFs of the five broad disease categories in PNG. In a country where most deaths occur outside of health facilities, verbal autopsy appears to be the only option to reduce ignorance about the causes of home deaths. The introduction of VA into selected districts in PNG as part of a broader effort by the Bloomberg Data for Health Initiative to improve cause of death data offers some optimism that this critical data gap can be addressed cost-effectively over the next few years [46]. The estimated cause specific mortality fractions by disease group and by province in this study should provide valuable health intelligence and reduce persistent knowledge gaps about causes of death in PNG.

## Supplementary information

**Additional file 1: Table S1.** Estimated CSMFs (%) by age and sex, DHIS and community, PNG, 2011. **Table S2.** Estimated CSMFs (%) by province and sex, PNG, 2011. **Table S3.** Composite index components by province, PNG. **Table S4.** Facility deaths as a percentage of all deaths, by province and sex, PNG, 2011. **Table S5.** Coefficients in the expected cause patterns method. **Figure S1.** Under-five mortality rate (per 1000 live births) by province, PNG, 2011 [10]. **Figure S2.** Male life expectancy by province, PNG, 2011 [10]. **Figure S3.** Female life expectancy by province, PNG, 2011 [10]

## Data Availability

Request for data access should be directed to the corresponding author and will be granted subject to approval by the National Department of Health and Papua New Guinea Institute of Medical Research.

## Abbreviations

COD: Cause of Death
CSMFs: Cause Specific Mortality Fraction
DHIS: Discharge Health Information System
eNHIS: Electronic National Health Information System
GBD: Global Burden of Disease Study
NCDs: Non-communicable diseases
NDoH: National Department of Health
NHIS: National Health Information System
PiHP: Partnership in Health Project
PNG: Papua New Guinea
VA: Verbal Autopsy
