# Supplementary material for: Estimating the pattern of causes of death in Papua New Guinea
Source: BMC Public Health. 2019 Oct 22;19:1322. doi: 10.1186/s12889-019-7620-5 (PMC6805633; doi:10.1186/s12889-019-7620-5)
Supplement: Supplementary file 1 — Additional file 1: Table S1. Estimated CSMFs (%) by age and sex, DHIS and community, PNG, 2011. Table S2. Estimated CSMFs (%) by province and sex, PNG, 2011. Table S3. Composite index components by province, PNG. Table S4. Facility deaths as a percentage of all deaths, by province and sex, PNG, 2011. Table S5. Coefficients in the expected cause patterns method. Figure S1. Under-five mortality rate (per 1000 live births) by province, PNG, 2011 [10]. Figure S2. Male life expectancy by province, PNG, 2011 [10]. Figure S3. Female life expectancy by province, PNG, 2011 [10] [file 12889_2019_7620_MOESM1_ESM.docx]

**APPENDIX**

**Table 4 Estimated CSMFs (%) by age and sex, DHIS and community, PNG, 2011**

| **Source/ method** | **Sex** | **Male (%)** | | | | **Female (%)** | | | |
| --- | --- | --- | --- | --- | --- | --- | --- | --- | --- |
|  | **Age Group** | **<5** | **5-44** | **45-64** | **65+** | **<5** | **5-44** | **45-64** | **65+** |
|  | **% Deaths in health facilities** | 60 | 33 | 32 | 9 | 41 | 35 | 16 | 4 |
| **DHIS** | Endemic Infections | 95 | 40 | 31 | 31 | 95 | 50 | 32 | 32 |
|  | Emerging Infections | * | 28 | 23 | 14 | * | 27 | 21 | 12 |
|  | Endemic NCDs | 4 | 18 | 33 | 41 | 4 | 17 | 37 | 43 |
|  | Emerging NCDs | * | 2 | 9 | 11 | * | 2 | 8 | 11 |
|  | Injuries | 1 | 11 | 4 | 3 | 1 | 5 | 2 | 2 |
| **Community** | Endemic Infections | 73 | 21 | 22 | 23 | 83 | 29 | 20 | 23 |
|  | Emerging Infections | * | 16 | 17 | 4 | * | 20 | 18 | 4 |
|  | Endemic NCDs | 20 | 18 | 31 | 44 | 15 | 24 | 34 | 42 |
|  | Emerging NCDs | * | 17 | 15 | 16 | * | 12 | 15 | 16 |
|  | Injuries | 7 | 28 | 15 | 13 | 2 | 16 | 13 | 14 |

* Due to low number of emerging infections and NCDs at ages less than five years, all infections and all NCDs were combined for this age group. The five causes for this age group in the empirical method cause were estimated using methods explained in this Appendix.

**Table 5 Estimated CSMFs (%) by province and sex, PNG, 2011**

|  | Male | | | | | Female | | | | |
| --- | --- | --- | --- | --- | --- | --- | --- | --- | --- | --- |
| **Province** | **Endemic Infections** | **Emerging Infections** | **Endemic NCDs** | **Emerging NCDs** | **Injuries** | **Endemic Infections** | **Emerging Infections** | **Endemic NCDs** | **Emerging NCDs** | **Injuries** |
| **PNG** | **31** | **10** | **29** | **16** | **13** | **35** | **14** | **27** | **15** | **8** |
| Western | 35 | 9 | 29 | 14 | 13 | 38 | 10 | 30 | 13 | 9 |
| Gulf | 39 | 13 | 23 | 10 | 14 | 44 | 14 | 22 | 10 | 10 |
| Central | 27 | 6 | 29 | 18 | 20 | 31 | 11 | 29 | 16 | 13 |
| National Capital District | 24 | 12 | 30 | 23 | 11 | 27 | 14 | 30 | 23 | 7 |
| Milne Bay | 40 | 8 | 28 | 14 | 10 | 41 | 10 | 28 | 14 | 7 |
| Oro | 34 | 11 | 31 | 15 | 10 | 38 | 13 | 28 | 14 | 7 |
| Southern Highlands /Hela | 33 | 12 | 29 | 13 | 12 | 38 | 13 | 28 | 13 | 9 |
| Enga | 22 | 11 | 30 | 17 | 20 | 29 | 17 | 28 | 12 | 14 |
| Western Highlands/Jiwaka | 22 | 9 | 36 | 19 | 14 | 26 | 13 | 34 | 18 | 10 |
| Simbu | 22 | 10 | 34 | 19 | 14 | 28 | 21 | 28 | 15 | 8 |
| Eastern Highlands | 26 | 9 | 32 | 17 | 17 | 31 | 15 | 29 | 14 | 11 |
| Morobe | 35 | 12 | 28 | 13 | 12 | 39 | 16 | 25 | 13 | 8 |
| Madang | 35 | 12 | 26 | 14 | 13 | 40 | 15 | 25 | 11 | 8 |
| East Sepik | 37 | 8 | 25 | 13 | 17 | 40 | 12 | 26 | 13 | 10 |
| Sandaun | 42 | 11 | 24 | 11 | 13 | 45 | 14 | 23 | 9 | 8 |
| Manus | 36 | 5 | 32 | 17 | 9 | 35 | 9 | 31 | 18 | 7 |
| New Ireland | 33 | 7 | 32 | 16 | 11 | 35 | 11 | 30 | 16 | 7 |
| East New Britain | 34 | 9 | 29 | 16 | 11 | 36 | 12 | 26 | 18 | 8 |
| West New Britain | 36 | 11 | 23 | 14 | 15 | 39 | 14 | 22 | 14 | 11 |
| Bougainville | 35 | 9 | 27 | 14 | 14 | 37 | 11 | 28 | 15 | 8 |

**Table 6 Composite index components by province, PNG**

| **Province** | **Education (%)** | **Economic (%)** | **Health (%)** | **Composite index (%)** |
| --- | --- | --- | --- | --- |
| National Capital District | 94 | 99 | 91 | 95 |
| East New Britain | 90 | 65 | 69 | 75 |
| New Ireland | 85 | 49 | 47 | 60 |
| Bougainville | 82 | 50 | 47 | 60 |
| Eastern Highlands | 22 | 77 | 78 | 59 |
| Morobe | 54 | 62 | 60 | 59 |
| Western Highlands / Jiwaka | 28 | 78 | 68 | 58 |
| Simbu | 28 | 62 | 82 | 57 |
| West New Britain | 72 | 53 | 44 | 56 |
| Milne Bay | 81 | 12 | 59 | 51 |
| Manus | 96 | 43 | 10 | 50 |
| East Sepik | 57 | 52 | 38 | 49 |
| Madang | 55 | 51 | 37 | 48 |
| Central | 65 | 34 | 38 | 45 |
| Oro | 72 | 24 | 34 | 43 |
| Western | 82 | 26 | 13 | 41 |
| Sandaun | 49 | 30 | 9 | 29 |
| Southern Highlands / Hela | 15 | 26 | 44 | 28 |
| Gulf | 47 | 18 | 15 | 27 |
| Enga | 25 | 25 | 25 | 25 |

**Table 7 Facility deaths as a percentage of all deaths, by province and sex, PNG, 2011**

| **Province** | **Male (%)** | **Female (%)** | **Both (%)** |
| --- | --- | --- | --- |
| *PNG* | *32* | *22* | *26* |
| Western | 34 | 23 | 28 |
| Gulf | 17 | 13 | 15 |
| Central | 16 | 10 | 13 |
| National Capital District | 56 | 38 | 45 |
| Milne Bay | 37 | 24 | 30 |
| Oro | 36 | 24 | 29 |
| Southern Highlands / Hela | 20 | 11 | 14 |
| Enga | 22 | 16 | 19 |
| Western Highlands / Jiwaka | 30 | 26 | 27 |
| Simbu | 26 | 19 | 23 |
| Eastern Highlands | 27 | 24 | 26 |
| Morobe | 33 | 22 | 27 |
| Madang | 34 | 23 | 28 |
| East Sepik | 24 | 19 | 22 |
| Sandaun | 21 | 16 | 18 |
| Manus | 39 | 28 | 33 |
| New Ireland | 44 | 30 | 36 |
| East New Britain | 38 | 24 | 31 |
| West New Britain | 49 | 30 | 38 |
| Bougainville | 34 | 22 | 27 |

**Figure 1 Under-five mortality rate (per 1,000 live births) by province, PNG, 2011**^10^

**
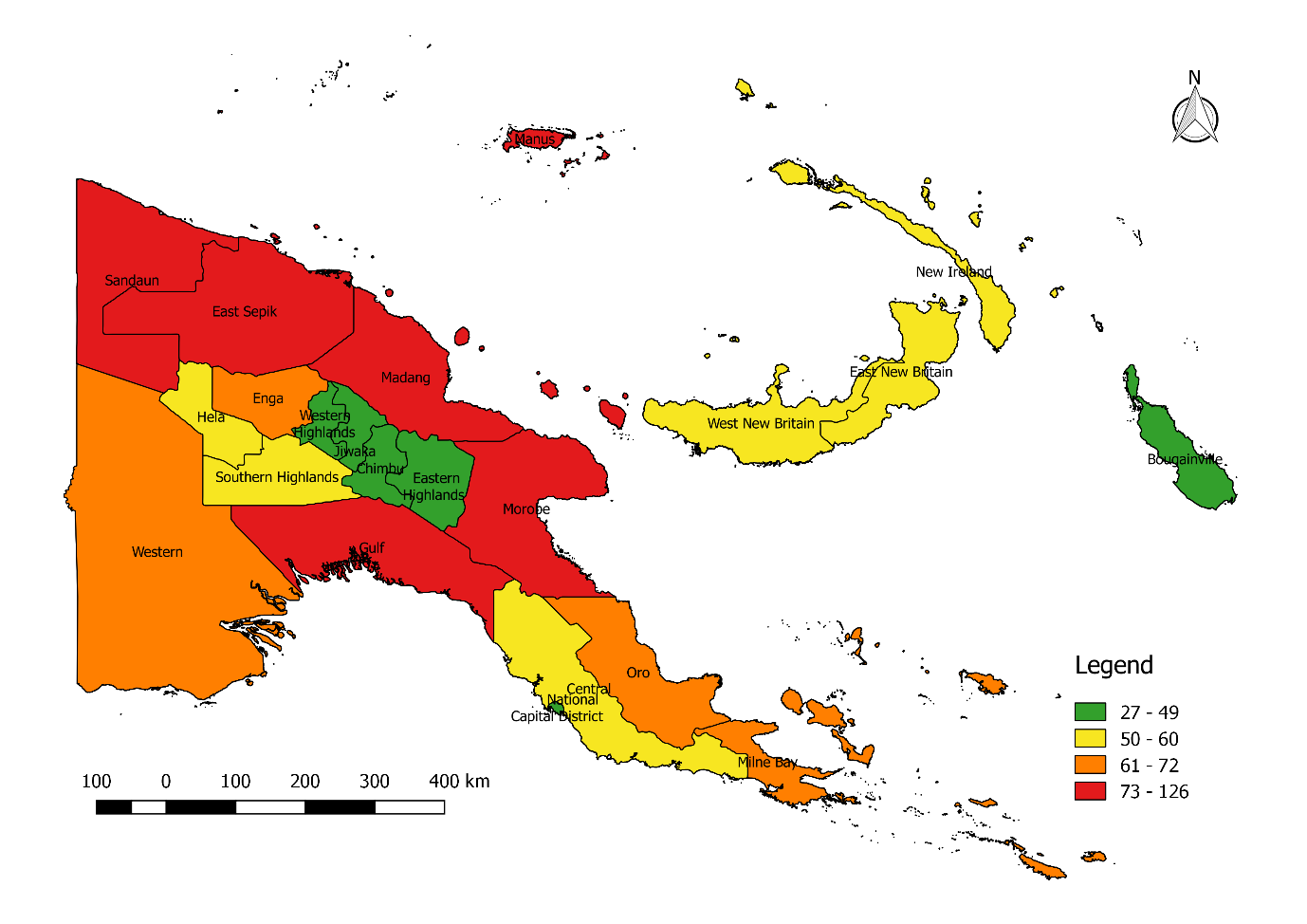
**

**Figure 2 Male life expectancy by province, PNG, 2011**^10^


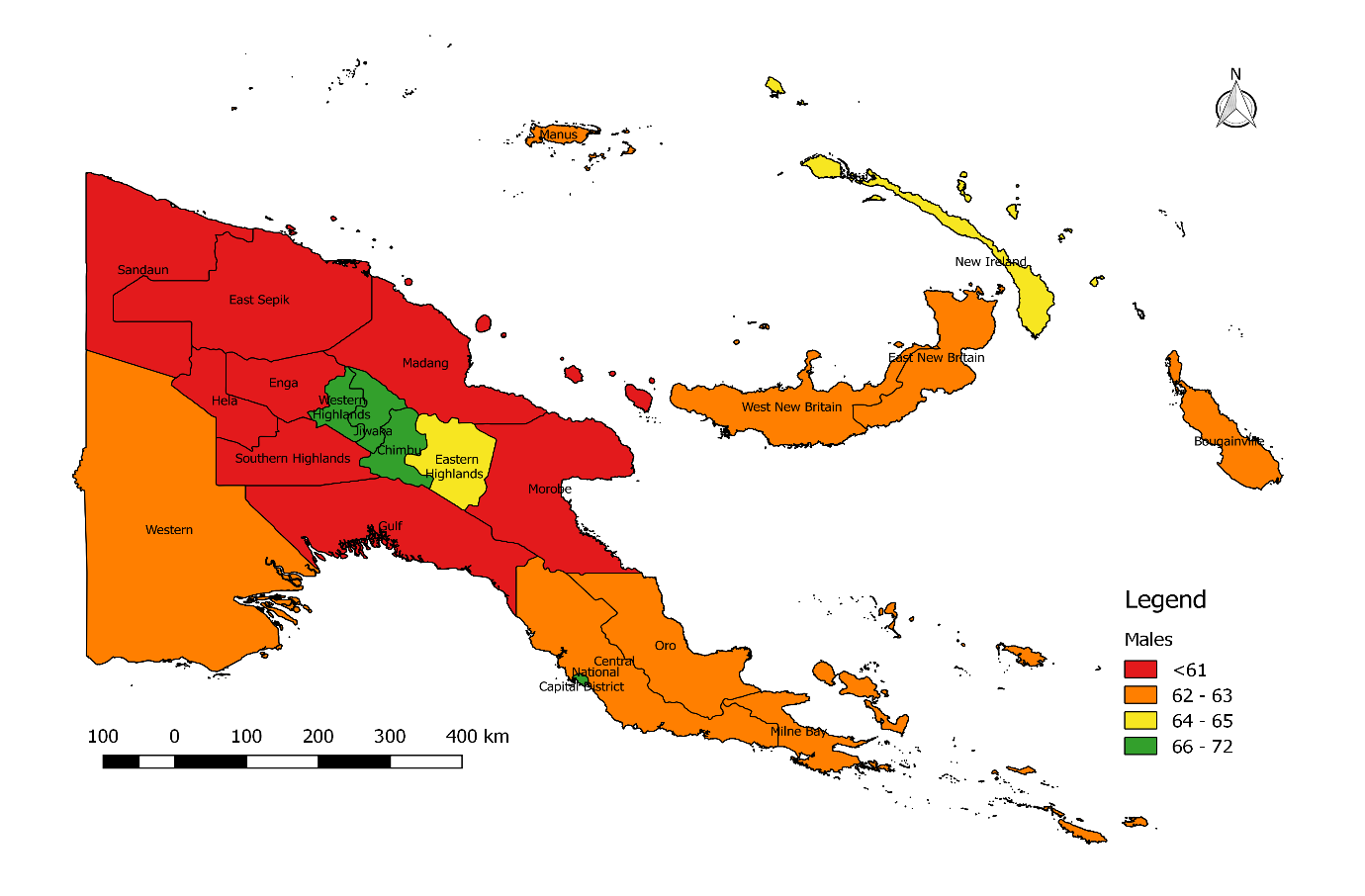


**Figure 3 Female life expectancy by province, PNG, 2011**^10^


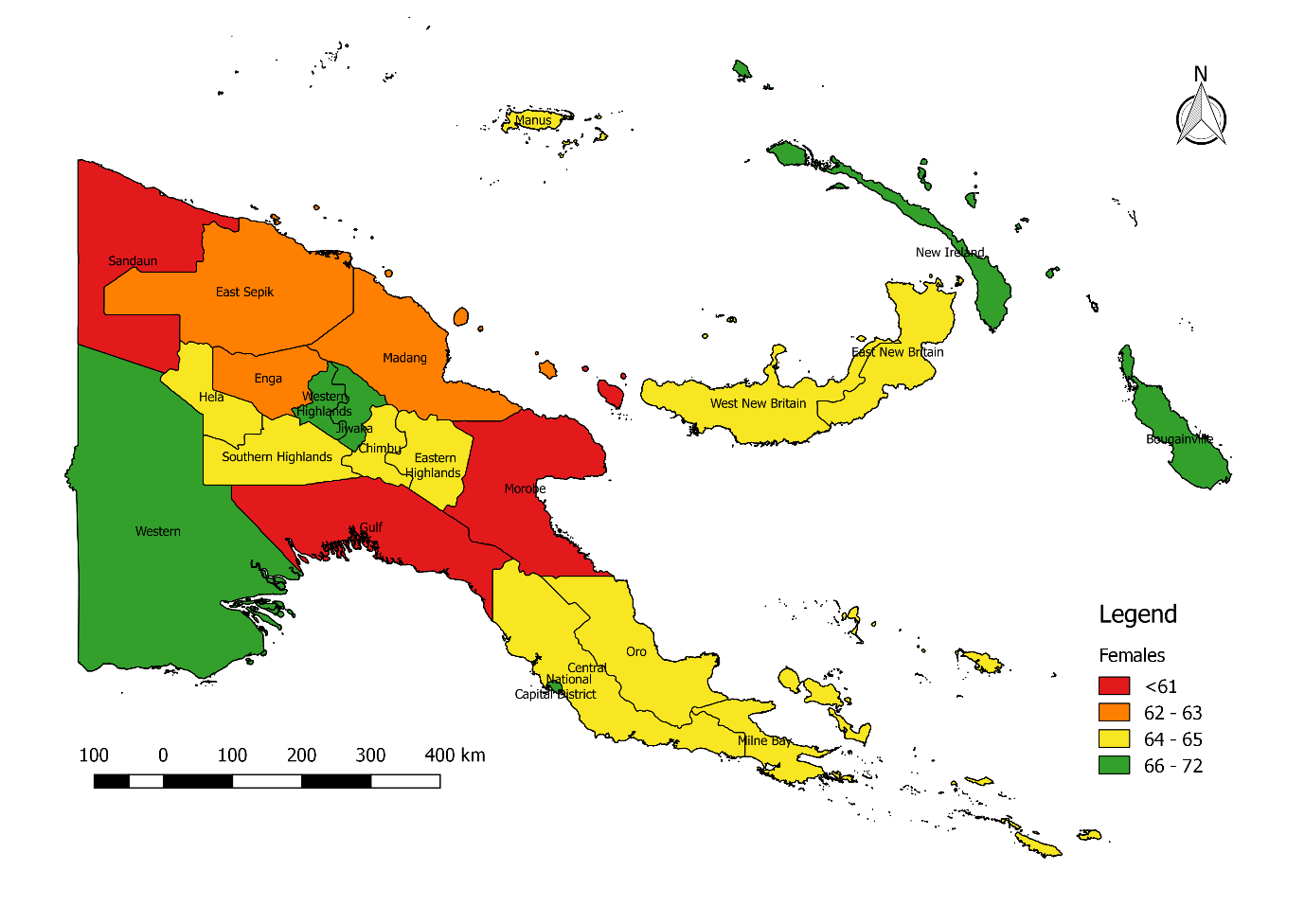


**Appendix**

***Empirical cause method***

*Estimating community CSMFs*

VA causes of death assigned as “Undetermined” are redistributed to specific causes of death using the Tariff algorithm. The redistribution is only conducted for three age groups (0-27 days, 28 days-11 years, 12+ years) which do not align with the age groups used in this study. In the community cause of death data, 18% of all deaths were assigned as Undetermined. CSMFs were compared before and after redistribution of Undetermineds, and the proportion of Undetermineds for each sex and in each of the three age groups that were re-assigned to each specific cause calculated. These proportions were then applied to causes of deaths in the four age groups used in our study (weighted within each age group) to redistribute Undetermineds to specific causes.

The ratios of VA to DHIS deaths in the four VA sites were calculated, in log space, as:

$${Ratio}_{asj}=\ln\left( \frac{{CSMF}_{VA,asj}}{{CSMF}_{VA,asz}} \right)-\ln\left( \frac{{CSMF}_{DHIS,asj}}{{CSMF}_{DHIS,asz}} \right)$$

where *a* is age group, *s* is sex, *j* is cause, and *z* is the base cause (Injuries). In this formula, the ratio of each of the other four causes to Injuries, for each age and sex grouping, is compared between the VA and DHIS. This is used to ensure that the final estimated CSMFs add to 100%. This approach is based on that used by Salomon et al (2003) and Rao et al (2009).

The ratio was then used to estimate community CSMFs in all provinces as follows:

$$\ln\left( \frac{{CSMF}_{COMM,aspj}}{{CSMF}_{COMM,aspz}} \right){=Ratio}_{asj}+\ln\left( \frac{{CSMF}_{DHIS,aspj}}{{CSMF}_{DHIS,aspz}} \right)$$

where *p* is province.

Next, for each cause *j* (using Cause 1 or Emerging Infections as an example):

$${CSMF}_{COMM,asp1}= \frac{e^{\ln\left( \frac{{CSMF}_{COMM,asp1}}{{CSMF}_{COMM,aspz}} \right)}}{e^{\ln\left( \frac{{CSMF}_{COMM,asp1}}{{CSMF}_{COMM,aspz}} \right)}+e^{\ln\left( \frac{{CSMF}_{COMM,asp2}}{{CSMF}_{COMM,aspz}} \right)}+e^{\ln\left( \frac{{CSMF}_{COMM,asp3}}{{CSMF}_{COMM,aspz}} \right)}+e^{\ln\left( \frac{{CSMF}_{COMM,asp4}}{{CSMF}_{COMM,aspz}} \right)}+1}$$

Then for cause *z* (or Injuries):

$${CSMF}_{COMM,aspz}=1-{CSMF}_{COMM,asp1}-{CSMF}_{COMM,asp2}-{CSMF}_{COMM,asp3}-{CSMF}_{COMM,asp4}$$

For the under 5 age group, insufficient deaths for some causes meant that the ratio was computed for all infectious diseases combined versus all other causes (i.e. all NCDs and injuries); these were later split using the proportions of the 5-cause categories within each broader cause group from the expected cause patterns method. Also, for some provinces at ages 65 years and over, insufficient deaths meant that the ratio for both sexes or even for all PNG was used.

*Combining community and DHIS CSMFs*

Before combining community and DHIS CSMFs, it was necessary to calculate the proportion of deaths in each province-age-sex grouping that occur in facilities and that occur in the community. (Table A4 shows the proportion of deaths in facilities as a proportion of all deaths by province and sex). Kitur et al ^10^ estimated life tables in 2011 for each province and sex. For these life tables, total deaths by province, broad age group and sex were calculated by multiplying the age-specific death rates for each five-year age group and multiplying the population for that five-year age group, and then aggregating the deaths into the broad age group. Next, the percentage of deaths that occurred in facilities was calculated by taking the maximum annual DHIS deaths in each facility by age and sex during 2007-13, summing these deaths for each age, sex and province, and then dividing by the total deaths for 2011.^^[[1]](#footnote-1)^^ The difference between this figure and 100% was the percentage of deaths that occurred in the community for each age, sex and province.

Finally, the CSMF for all deaths by age, sex and province was calculated as:

$${{CSMF}_{ALL,aspj}=CSMF}_{COMM,aspj}*d_{COMM,aspj}+{CSMF}_{DHIS,aspj}*d_{FACIL,aspj}$$

where *j* refers to each of the 5 cause categories, and *d* is the percentage of all deaths.

**Expected cause patterns method**

The model used to estimate age-sex-province specific CSMFs from GBD data is:

$$\ln\left( \frac{{CSMF}_{ascj}}{{CSMF}_{ascz}} \right)=ln\left( {{}q}_{asc} \right)+y+S_{cy}$$

where *a* is age group, *s* is sex, *j* is cause, and *z* is the base cause (in this model endemic NCDs), *c* is country, *q* is the probability of dying from the beginning to the end of the age group (equivalent to the *_n_q_x_* value using standard life table notation; e.g. for 5-44 it is the probability of dying from exact age 5 to exact age 45), *y* is calendar year and *S* is the SDI for the country. To estimate CSMFs for PNG, *q* values from the country life table developed in Kitur et al ^10^ were used. For each cause (*j*=1, 2, 3, 5), age group (0-4, 5-44, 45-64, 65+ years) and sex, a model was developed. This was used to predict $ln\left( \frac{{\dot{\hat{CSMF}}}_{asj}}{\hat{CSMF}_{asz}} \right)$ for each cause *j* in PNG, where $\dot{\hat{CSMF}}$ is the predicted CSMF for PNG.

Next, for each cause *j* (using cause 1 or Emerging Infections as an example):

$$\hat{CSMF}_{as1}= \frac{e^{\ln\left( \frac{{\dot{\hat{CSMF}}}_{as1}}{\hat{CSMF}_{asz}} \right)}}{e^{\ln\left( \frac{{\dot{\hat{CSMF}}}_{as1}}{\hat{CSMF}_{asz}} \right)}+e^{\ln\left( \frac{{\dot{\hat{CSMF}}}_{as2}}{\hat{CSMF}_{asz}} \right)}+e^{\ln\left( \frac{{\dot{\hat{CSMF}}}_{as3}}{\hat{CSMF}_{asz}} \right)}+e^{\ln\left( \frac{{\dot{\hat{CSMF}}}_{as5}}{\hat{CSMF}_{asz}} \right)}+1}$$

Then for cause *z* (cause 4 or endemic NCDs):

$$\hat{CSMF}_{as4}=1-\hat{CSMF}_{as1}-\hat{CSMF}_{as2}-\hat{CSMF}_{as3}-\hat{CSMF}_{as5}$$

Once CSMFs for each age and sex in PNG were calculated, a model to estimate provincial level CSMFs was developed. In PNG, the absence of reliable provincial level fertility data means that an equivalent of the SDI cannot be used. Therefore, a model that relied only on all-cause mortality to predict variation in CSMF across provinces was also developed from the GBD 2017 data for 195 countries and territories. This model is:

$$\ln\left( \frac{{CSMF}_{ascj}}{{CSMF}_{ascz}} \right)=ln\left( {{}q}_{asc} \right)+y$$

This model predicts $ln\left( \frac{{\dot{\hat{CSMF}}}_{aspj}}{\hat{CSMF}_{aspz}} \right)$ for each age, province *p* and cause *j*. Calculations for each cause were conducted as above. The last step was to scale the CSMFs for each province to those for all PNG. This was done, for each age-sex-cause grouping, by:

1. Adjusting the provincial CSMF for each age, sex and cause to the national age, sex and cause as estimated by the model above.
2. Adjusting the adjusted provincial CSMF for each age, sex and cause so that the total within each age-sex-province group adds to 100%.

Coefficients for the expected cause patterns method are shown below.

**Table 8 Coefficients in the expected cause patterns method**

|  | **Males** | | **Females** | |
| --- | --- | --- | --- | --- |
| ***Less than 5 years*** | **Coefficient** | **p-value** | **Coefficient** | **p-value** |
| *ln (emerg. inf./end. NCD)* |  |  |  |  |
| Probability of dying | 1.406 | 0.000 | 1.352 | 0.000 |
| Year | 0.035 | 0.000 | 0.039 | 0.000 |
| Constant | -67.740 | 0.000 | -76.276 | 0.000 |
| *ln (end. inf./end. NCD)* |  |  |  |  |
| Probability of dying | 0.693 | 0.000 | 0.684 | 0.000 |
| Year | 0.009 | 0.000 | 0.009 | 0.000 |
| Constant | -15.309 | 0.000 | -13.578 | 0.000 |
| *ln (emerg. NCD/end. NCD)* |  |  |  |  |
| Probability of dying | 0.262 | 0.000 | 0.166 | 0.000 |
| Year | -0.012 | 0.000 | -0.016 | 0.000 |
| Constant | 21.226 | 0.000 | 28.466 | 0.000 |
| *ln (injuries/end. NCD)* |  |  |  |  |
| Probability of dying | 0.227 | 0.000 | 0.226 | 0.000 |
| Year | -0.006 | 0.000 | -0.004 | 0.000 |
| Constant | 10.756 | 0.000 | 7.204 | 0.000 |
|  | **Males** | | **Females** | |
| ***5-44 years*** | **Coefficient** | **p-value** | **Coefficient** | **p-value** |
| *ln (emerg. inf./end. NCD)* | 2.008 | 0.000 | 1.512 | 0.000 |
| Probability of dying | 0.021 | 0.000 | 0.028 | 0.000 |
| Year | -38.949 | 0.000 | -52.360 | 0.000 |
| Constant |  |  |  |  |
| *ln (end. inf./end. NCD)* | 1.394 | 0.000 | 1.373 | 0.000 |
| Probability of dying | 0.003 | 0.040 | 0.003 | 0.010 |
| Year | -3.170 | 0.229 | -2.245 | 0.289 |
| Constant |  |  |  |  |
| *ln (emerg. NCD/end. NCD)* | -0.217 | 0.000 | 0.041 | 0.000 |
| Probability of dying | -0.009 | 0.000 | -0.007 | 0.000 |
| Year | 17.374 | 0.000 | 12.347 | 0.000 |
| Constant |  |  |  |  |
| *ln (injuries/end. NCD)* | 0.070 | 0.000 | 0.085 | 0.000 |
| Probability of dying | -0.008 | 0.000 | -0.010 | 0.000 |
| Year | 16.215 | 0.000 | 19.153 | 0.000 |
| Constant | 2.008 | 0.000 | 1.512 | 0.000 |
|  | **Males** | | **Females** | |
| ***45-64 years*** | **Coefficient** | **p-value** | **Coefficient** | **p-value** |
| *ln (emerg. inf./end. NCD)* | 2.788 | 0.000 | 1.914 | 0.000 |
| Probability of dying | 0.013 | 0.000 | 0.020 | 0.000 |
| Year | -24.549 | 0.000 | -38.780 | 0.000 |
| Constant |  |  |  |  |
| *ln (end. inf./end. NCD)* | 1.588 | 0.000 | 1.840 | 0.000 |
| Probability of dying | 0.009 | 0.000 | 0.013 | 0.000 |
| Year | -17.691 | 0.000 | -25.445 | 0.000 |
| Constant |  |  |  |  |
| *ln (emerg. NCD/end. NCD)* | -0.009 | 0.469 | 0.376 | 0.000 |
| Probability of dying | -0.007 | 0.000 | -0.004 | 0.000 |
| Year | 14.742 | 0.000 | 9.007 | 0.000 |
| Constant |  |  |  |  |
| *ln (injuries/end. NCD)* | 0.176 | 0.000 | 0.303 | 0.000 |
| Probability of dying | -0.002 | 0.006 | -0.003 | 0.000 |
| Year | 2.672 | 0.046 | 3.547 | 0.011 |
| Constant | 2.788 | 0.000 | 1.914 | 0.000 |
|  | **Males** | | **Females** | |
| ***65+ years*** | **Coefficient** | **p-value** | **Coefficient** | **p-value** |
| *ln (emerg. inf./end. NCD)* | 6.957 | 0.000 | 3.692 | 0.000 |
| Probability of dying | 0.004 | 0.128 | 0.011 | 0.000 |
| Year | -8.608 | 0.065 | -23.163 | 0.000 |
| Constant |  |  |  |  |
| *ln (end. inf./end. NCD)* | 2.355 | 0.000 | 2.249 | 0.000 |
| Probability of dying | 0.004 | 0.004 | 0.009 | 0.000 |
| Year | -9.701 | 0.001 | -18.254 | 0.000 |
| Constant |  |  |  |  |
| *ln (emerg. NCD/end. NCD)* | 0.575 | 0.000 | 0.687 | 0.000 |
| Probability of dying | -0.005 | 0.000 | -0.004 | 0.000 |
| Year | 10.222 | 0.000 | 9.343 | 0.000 |
| Constant |  |  |  |  |
| *ln (injuries/end. NCD)* | 1.005 | 0.000 | 0.759 | 0.000 |
| Probability of dying | -0.001 | 0.335 | -0.001 | 0.332 |
| Year | -0.716 | 0.611 | -0.922 | 0.544 |
| Constant | 6.957 | 0.000 | 3.692 | 0.000 |

Emerg.: emerging. End.: endemic.

1. The maximum deaths was chosen to overcome problems with incompleteness of reporting of deaths in facilities. [↑](#footnote-ref-1)
